# Using unfolding case studies to develop critical thinking for Graduate Entry Nursing students: an educational design research study

**DOI:** 10.1186/s12912-024-02076-8

**Published:** 2024-06-11

**Authors:** Rachel Macdiarmid, Eamon Merrick, Rhona Winnington

**Affiliations:** 1 Department of Nursing, Faculty of Health and Environmental Science, Auckland University of Technology, 90 Akoranga Drive, Northcote, Auckland, 0627 New Zealand; 2 Nursing and Midwifery Directorate NSLHD, Royal North Shore Hospital, Level 7 Kolling Building, St Leonards, NSW 2065 Australia; 3 Nursing Faculty of Health, University of Technology Sydney, Sydney, Australia

**Keywords:** Graduate entry nursing, Nursing education, Problem-based learning, Students, Unfolding case-study, Experiential pedagogy

## Abstract

**Background:**

Graduate Entry Nursing (GEN) programmes have been introduced as another entry point to nurse registration. In the development of a new GEN programme, a problem-based approach to learning was used to develop critical thinking and clinical reasoning skills of motivated and academically capable students.

**Objective:**

To explore and evaluate the design and delivery of course material delivered to GEN students embedded in authentic learning pedagogy from the perspectives of both GEN students and academic staff using an unfolding case study approach.

**Methods:**

An educational design research approach was used to explore the learning experiences of GEN students using an unfolding case study approach situated in experiential pedagogy and the teaching experiences of the academics who designed it. Data were collected through semi-structured interviews with students once they had finished the course and weekly reflective diary recordings by academic staff throughout implementation. Thematic analysis was used to analyse the data.

**Findings:**

Student reflections highlighted that this cohort had insight into how they learned and were comfortable voicing their needs to academic staff. While the unfolding case studies were not liked by all participants, for some it offered a unique learning opportunity; particularly when scaffolded with podcasts, simulation labs, tutorials and clinical placements. Staff reflections primarily aligned with student experiences.

**Conclusion:**

The gaps highlighted in the delivery of the course suggest that a blended pedagogical approach to graduate entry nurse education is required. Specifically, GEN students are aware of the learning needs and are happy to express these to academic staff, thus suggesting that engaging with a co-design curriculum approach will benefit future cohorts.

**Supplementary Information:**

The online version contains supplementary material available at 10.1186/s12912-024-02076-8.

## Problem

Graduate entry nursing students begin their degrees as experienced learners and must develop critical thinking skills within the shortened degree time frame.

## What is already known

Graduate entry students are experienced and academically capable learners who begin with a diverse range of life and career experiences.

## What this paper adds

Graduate entry students would benefit by being involved in curriculum design to acknowledge the unique skill set that they bring.

## Introduction

Graduate Entry Nursing (GEN) degrees, or second degrees leading to eligibility for nursing registration, have recently been introduced to New Zealand. GEN students are known to be academically capable, motivated, and driven, bringing with them a range of life experiences, and have often had significant careers before enrolment [1, 2]. Previous research has identified that teaching and learning methods must be carefully planned and innovative [1].

Pre-registration nursing education programmes prepare nursing students to provide safe nursing care with crucial skills expected of nursing graduates, including critical thinking and clinical reasoning. Clinical reasoning enables students to approach clinical issues with a problem-solving lens that relies on gathering assessment data and intervening and evaluating the patient’s response to the intervention [3].

Problem-Based Learning (PBL) aligns with the fundamental elements of authentic learning approaches [4], where learning is situated in real-world contexts [5]. Problem-based learning is considered to be an experiential teaching and learning approach that helps students develop a critical lens and clinical reasoning skills [6, 7]. The use of PBL in nursing education is well established with previous research focused on students’ experiences and satisfaction [8]; factors that facilitate or hinder students' learning [9]; and the development of critical thinking skills [10].

Graduate entry nursing students report enjoyment of the active learning sets that enabled discussion surrounding case studies, scenarios, and practice issues [11]. Cangelosi’s [12] phenomenological study found that although time-poor, GEN students welcomed learning opportunities that were not traditional and facilitated their development and growth.

However, there is conflicting evidence regarding the effectiveness of PBL in nursing. For example, McCormick et al. [13] compared undergraduate student performance using differing teaching approaches, such as unfolding simulation scenarios versus recorded lectures and found these to be of benefit to students. Carter and Welch [14] compared the results of associate degree nursing students who attended lectures to those whose learning was informed by an unfolding case study. In contrast to McCormick’s et al.’s [13] earlier positive results, these authors found both groups of students performed worse in the post-test.

As previous research has identified that new graduate nurses do not always have critical thinking skills, using an unfolding case study approach can reflect the reality of clinical practice where not all the relevant information is known at the first encounter with the patient [14–16].

Nonetheless, while several studies have investigated the use of unfolding case studies in undergraduate preregistration programmes there is little evidence that supports the use of these with more academically capable GEN students. This article reports on a qualitative interpretivist study that used an educational design methodology to explore the experiences of GEN students who participated in the programme of learning and the experiences of the academics who designed it.

## Methods 

Educational Design Research (EDR) is an iterative, pragmatic, and reflective methodology well suited to small projects [17]. It has arisen from design-based research and can include both quantitative and qualitative data collection methods. EDR was selected as it fitted with our desire to develop new ways of teaching alongside gaining feedback from both academic staff and students. In the first phase of this research, we redesigned the teaching and learning strategies for a component of the GEN programme [18].

EDR has four phases (Table 1) [17]:

**Table 1:** Phases of EDR

Phases of Educational Design Research		
1	Analysis and exploration	
2	Design and construction: Designing the learning environment, delivering content and facilitation of learning experiences designed in phase one	Iterative redesign and re-evaluation
3	Evaluation and reflection: inclusive of academic reflections and student feedback	
4	Theory building: further development and dissemination of the findings	

### Aims and objectives

The study aimed to explore and evaluate the design and delivery of course material delivered to GEN students embedded in authentic learning pedagogy from the perspectives of both GEN students and academic staff using an unfolding case study approach.

### Theoretical framework

To enable the development of clinical reasoning skills a scaffolded learning approach was implemented that involved unfolding case studies designed to represent the health needs of the New Zealand population, thus, encouraging critical thinking. Unfolding case studies reflective of situations that students might face in the future were used to encourage students to consider and analyse information, provoke further questioning and identify the information required to narrow their inquiries [14, 15]. Supported by this evidence the academic staff built a learning environment where a regular teaching schedule (two days of lectures and one day of clinical labs per week), was complemented with online resources. Initial questions about the case study were provided on the learning management system. Students attended simulations where they responded to the case and answered questions critical to unpacking the ‘patients’ reality. Alongside the unfolding case studies were podcasts where experts were interviewed on topics related to the case. Tutorials enabled students to collaboratively construct answers and share their perspectives; at the end of each week students shared their answers in an online discussion forum.

### Methods and setting

This study was conducted at an education facility in New Zealand offering undergraduate and GEN programmes. The participants are academics involved in the design and delivery of the course and one cohort of students of the GEN programme. This article reports on Phase 2 and 3 of the EDR approach, the academic staff’s reflective diary during course delivery, and students' feedback after the course was completed the first time. The methods were reported using the Consolidated Criteria for Reporting Qualitative Studies (COREQ) [19].

### Participants

Purposeful sampling was used as the researchers were keen to explore the experiences of a specific GEN cohort [20]. Academic staff involved in the weekly reflective diaries are also the research team (*n* = 3). All students in the identified cohort (*n* = 7) were invited to participate, totalling ten possible participants. Student participants were approached via an advertisement on the university’s learning management system. Students were asked to contact the research assistant, who was separate from the academic staff and was not involved in the delivery of the GEN programme; five students agreed to participate. A $20 petrol voucher was offered to those who participated.

### Data collection and analysis

In keeping with education design methodology, the authors met weekly to reflect on their experiences of delivering the content and guiding students. The weekly reflective conversations, between 60–90 min in length, followed a simple format of ‘what worked, what didn’t work, and what would we (as academic staff) change?’ Face to face student interviews were conducted by the research assistant at a time and place convenient to the students using semi-structured questions that were developed by the research team (see Additional file 1).

The semi-structured interviews (*n* = 5) and reflective meetings (*n* = 9) were recorded and transcribed verbatim by a research assistant who had signed a confidentiality agreement. All identifying information was deleted from the transcripts by the research assistant before the research team reviewed the data; each recording and transcript was allocated a unique identifier, for example ‘participant one’.

Thematic analysis [21, 22] was used to analyse the data. First, the research team independently read the transcribed interviews to familiarise themselves with the data and identified initial codes. Second, the researchers met and reviewed all transcripts to identify themes and reached consensus on the themes emerging from the data. Themes were established once more than 50% of the participants stated the same issue/thought/perception. A matrix was developed whereby common themes were identified, with quotes demonstrating the themes collated to establish an audit trail.

### Reflexivity

Central to this study given the proximity of staff to this student cohort, a reflexive stance was essential. Reflexivity is an engendered practice and was used in this instance not to influence the direction and outcome of the research but to allow the researchers to engage in the data to produce viable and valuable outcomes for future staff and students. Specifically, this reflexive practice provided a means for the research to be rigorous through the consideration of the vulnerability of the participating student cohort, thus inciting reflection-before-action [23].

### Ethical considerations

Ethical approval for this study was obtained from the Auckland University of Technology Ethics Committee (AUTEC) (19/233). Given the potential power differential in the student/staff relationship present, participants were approached via an online advertisement and followed up by an independent research assistant. This is key to the success of the project, as such research undertakings have the potential for conflict of interest to exist [24]. The academic staff recordings were also undertaken with the knowledge that these would remain confidential to the participants and transcriber only, with a memorandum of understanding completed to this effect. Participant information sheets were given to students interested in joining the study to ensure they knew what it entailed and how their safety and identity would be managed. Written consent was obtained before the interviews were undertaken, with oral consent obtained at the beginning of each interview.

## Results

Three dominant themes emerged, which focused on the experiences of both GEN students and teaching staff. These were:

1. Reflective learning: Students and staff ability to clarify what worked and what did not work
2. Evaluation of learning: Students and staff being insightful about their ways of learning and needs
3. Challenges: Planning and delivering appropriate content for GEN students is challenging for teaching staff.

Within these overarching themes, subthemes were developed and will be presented in the following data results (Table 2).

**Table 2:** Themes and subthemes

Themes	Reflective learning	Evaluation of learning	Challenges
Subthemes	• Unfolding case study as problem-based approach • Use of podcasts	• Approaches to learning • Students as insightful and engaged	• Teaching staff experiences of GEN student learning

### Reflective learning

The exploration of student and staff experiences and responses to the unfolding case studies unearths what worked and what was problematic for both parties.

### Unfolding case study as problem-based approach

The student experiences of using an unfolding case study approach were divided. Some students enjoyed the case scenarios but did not necessarily find them beneficial in terms of knowledge advancement as.

“*I personally, like the case studies but personally I didn’t really find that they enhanced my learning in like the clinical setting*” (P1)

or that they were relevant to clinical practice in that.

“…*some of it was definitely relatable but I just found it was very different in the clinical setting compared with doing this theoretical case setting*” (P1).

A second student supported this idea that the case studies did not add practical clinical knowledge value as.

“*I mean for me the case studies weren’t challenging…I didn’t think the case studies added anything extra into my practice, they didn’t challenge my clinical reasoning or anything like that*” (P2).

Of note was that those students with previous professional healthcare backgrounds found the use of an unfolding case study approach problematic in that.

“*I found that quite a challenge. I think because with my clinical background I was sort of going straight into, yeah like I wanted more information so you know I probably would have preferred…to have a different case study every week or have all the information…and I’d be like well what about this, what about that?*” (P5).

Participant One, however, noted that while the case studies may not have added knowledge value, they were helpful at times as.

“*…one example is we learnt about arterial blood gases and then I was on placement I came across that literally [on] day one, so was really nice to be able to put something that I’d learnt in class into practice*” (P1).

While some students were less keen on the case study approach and found them hard work, others thought they provided opportunities to encourage discussion, clinical reasoning, and autonomous thinking as.

“*there was no right or wrong answer, you just had to prove your point to say I think it is this because of this, and someone else can say something else and just kind of still prove it because it was a quite grey [area] but I actually found that it really got us thinking*” (P3).

Moreover, the same participant acknowledged that.

*“…I think that’s the whole idea of the course [GEN Programme] because at this level they shouldn’t be spoon-feeding you…you should be able to think for yourself and reason things out*” (P3).

Although some discord was present with regard to the case study approach, one participant did acknowledge the value of being able to break down a huge scenario into manageable sections to enhance understanding and clinical decision-making, as.

“*when you break it down it makes it easier to kind of work out what you’re going to do and what steps you’re going to do*” (P4), and that “*because you start looking at the smaller things that you need to do rather than just the big bits*” (P4).

It appears, however, that staff involved in the programme of learning were pleased with the overall notion that problem-based learning approach offered a ‘practical’ means through which to discuss what is the hands-on job of nursing. Specifically,

“*the second session around child abuse and recognising child abuse…took me a bit by surprise as I wasn’t expecting that to go very well and it went extraordinarily well, mostly because it was case based again and story based*” (L1).

Moreover, with regard to encouraging discussion and clinical reasoning at a postgraduate level,

“*I think we’ve really pulled out the difference [of] what we’re expecting of them [GEN students] as opposed to what they may have been used to”* (L1).

### Use of podcasts

While the use of technology is not necessarily a completely new strategy in tertiary education, here we have linked podcasts recorded with experts in their fields which related to the unfolding case studies, Again, however, there was division in the value of podcast recordings, with some students really enjoying them, saying.

“*I liked the podcasts yeah, I found the podcasts really good especially when there was [sic] different people talking about it, yeah...podcasts are good, like to just chuck on in the car or at the gym*” (P2).

Moreover, some found them easy to listen to because.

“…*it’s a different way to learn because like you’ve got YouTube videos and you’ve got books and stuff but podcasts are kind of like easy*” (P2).

Some students found the podcasts particularly engaging saying.

*…I just remember listening to it and I think I was in the car and I had stopped because I was on my way home…and I was still listening to it in the garage like when I was home and I was like oh this is a really interesting podcast*” (P2).

Participant three also thought podcasts a positive addition to the resources saying.

“*yeah they were helpful…there was one I listened to…they were talking about dying…I know that [one of the lecturers’] kind of research is kind of talking about death, euthanasia and all this kind of thing, and for some reasons, I don’t know why, maybe that’s why I still remember, I can say it’s the only podcast I really listened to and it was really good because it gave me a good insight as to what is happening…*” (P3)

This positive response was also noted in face-to-face class time as one staff member reported that.

“*they* *[the students] loved the person who was interviewed, and the feedback was it was really nice to hear a conversation about different perspectives*” (L1).

Yet, not all students were of this opinion, with some advising the podcasts were too long (approximately 60 min each), that they can be distracting, that they preferred videos and images or an in-person discussion, saying.

“*I find podcasts…I tend to switch off a bit, a bit quicker than if I was watching something, I would probably prefer, rather than watching a podcast [sic] I’d rather have an in-class discussion with the person”* (P4).

Participant one said that they too struggled with podcasts because.

“*I’m more visual so I like to look at things and see like a slide I guess or what they’re talking about or, so I sort of zone out when it’s just talking and nothing to look at, so that’s what I personally struggle with, they [podcasts] are helpful it’s just I’m more a visual learner*” (P1).

While there were some negative responses to the podcasts, another participant acknowledged their value but offered their own solutions to learning, saying that.

“*I listened to a few podcasts that were put up, because they’re just easy to listen to*” (P2).

but felt that overall there were insufficient resources made available to students and therefore.

“*just went to YouTube and just, any concepts that I was unfamiliar with or stuff in class that we went over and when I went home I was like [I have] no idea what they talked about, I just found my own videos on YouTube…*” (P2).

### Evaluation of learning 

Learning experiences are unique to each GEN student, as are those experienced by the teaching staff. The data collected highlighted this clearly from both perspectives, offering a particularly strong insight into how this cohort of students’ function.

### Approaches to learning

It was evident that these GEN students were aware of their approach to learning and that perhaps the structure of the teaching module did not align with their needs as.

“*I’m not really the best at utilising online things I’m a really hands on learner and things like a lecture…but you know if it’s yeah, more like class time, it’s sort of more my, my learning style [I] guess*” (P5).

A number of students were able to identify that they were visual learners as.

“*I use videos more because I guess I’m more of a visual learner as well and I learn better by seeing things instead of reading a huge article, I think that [videos] it helps me a bit more”* (P4).

Another student, however, preferred a discussion based approach as opposed to either videos or podcasts saying that.

“*if it’s interesting, if it’s a topic that you can like relate to [through a podcast] or something it’s fine,* *but for me I just switch off not really taking a lot of the information [in] whereas in a discussion setting you can ask questions and you can interact with the person, yeah I find that would be a bit more helpful*” (P4).

This approach to learning through discussion was also noted when the teaching staff reflected on their experiences in that in one teaching session the GEN students.

“*were engaged, they were round a table with the second speaker talking and what I think enabled the discussion was that she [the speaker] was using her data as stories and so she was reading them, actually she got them [the students] to read them out”* (L3).

The notion of learning styles, however, was not as linear as being visual or auditory or practical, as one student noted that a combination of styles was preferable to enhance learning, saying that.

“*if* *we weren’t able to have lectures like a recorded lecture so that there was a PowerPoint and just someone actually talking you through it, like I know there’s the YouTube videos…some of them were a little bit helpful, but like I just felt that sometimes we missed the teaching aspect of it. There’s a lot of self-directed stuff but definitely like a recorded lecture every week to go along with the readings and extra videos to watch*” (P5).

### Students as insightful and engaged

While GEN students are known for their tenacity and ability to cope with the pressure and fast paced delivery, some students discovered that this did not necessarily equate with their preferred approach to learning. This cohort of GEN students were insightful in terms of their strengths and weaknesses in relation to knowledge acquisition. The use of the unfolding case studies, however, caused some frustrations as.

“*for me it was challenging in the fact that I felt I actually got frustrated because I’m thinking well I want to know this, I want to know that and yeah not getting all the information that I wanted at the time*” (P5).

This participant went further, saying that.

“*I definitely found that difficult [lack of information] I felt like [I] wasn’t getting as much information as I wanted to be able to make my clinical decisions*” (P5),

however this may have been due to the student’s background as their.

“my *background is in paramedicine*” where “*we get a lot of information in a very short amount of time*” (P5).

Some fundamental issues were raised by the participants in terms of how much study is required for them to acquire the new knowledge. As one student highlighted,

“*I have a really terrible memory, so I kind of need to listen to things a few times or write it down and then watch a video and do some more reading and then like it’s good having another element to get into your brain you know*” (P2).

For one student, a solution to this was to ensure they did their preparation before attending class as.

“*you’re supposed to have read these things before coming to class, some people don’t but my kind of person, I’d read before coming to class and I tended to answer those questions so the critical, analytical part of me would be trying to find out and come up with a reasonable answer…”* (P3).

For another participant, they took an alternative pathway to learning as they.

“*I just watch it and I don’t take [it in], it just sits in the back of my head because sometimes it’s building on top of previous knowledge so just, I just watch it to see if I can gain anything from* *that, I don’t necessarily take down notes or anything, but I just watch it so that it’s there you know*” (P4).

The pace of content delivery appeared problematic for some students, especially in relation to the practical sessions, with one student highlighting that.

“*personally I didn’t’ really like it and most of the time they were rushing, I was always like can I write this down to go back home to like really make sense of it and then sometimes obviously, sometimes I would have to say can I stay back and practice this thing again [as] I didn’t grab it as quickly as others did and the essence of the labs is that it’s grab all of these things*” (P3).

### Challenges: Teaching staff experiences of GEN student learning

While on the whole the teaching staff were able to gauge the learning needs of this GEN cohort, the expectations of both parties did not always align, with one staff member reporting that.

“*the* *two biggest challenges was [sic] getting them [the students] to unpack already learned behaviour and [to] acknowledge their own limitations or bias*” (L1),

however by the end of the semester the same staff member reported that.

“*I think we made a lot of progress in getting them to acknowledge how they learn*” (L1).

Moreover, the challenges anticipated in teaching GEN students were not those that transpired in that.

“*I actually thought going into the first paper I was pretty excited as to how it was going to roll* *out,* *the problems I encountered were not the problems I anticipated*” (L3).

The vocality of this cohort was tangible, however, when content did not meet their needs, interest or expectations with the students saying,

“*that they didn’t do the materials because it wasn’t of interest to them and requested other teaching very much related to the assignment as opposed to anything else*…” (L1).

It was expected that the GEN students would be participatory both in class and online irrespective of their ways of learning, but there was a difference in both responses and comfort with this form of engagement. One student that talked about the unfolding case study and the online component of assessment as being problematic said that.

“..*we had to put up about 250 words of something related to the case study every week and then we spoke to someone else, [I] didn’t really like the responses…I didn’t really like having to respond to someone else*” (P3).

Yet in contrast to this statement, the teaching staff were delighted that.

*“…actually I got some fantastic questions from one of the students…emailed to me on Monday night about the case that was online for them, questions that I didn’t talk about in [the] lecture, I didn’t introduce the concept…they’re talking about concepts that are currently undergoing international clinical trials”* (L1).

## Discussion

This study explored the experiences of both GEN students and academics using unfolding case studies situated in experiential learning pedagogy. The use of unfolding case studies supported with podcasts embraced our idea of developing content situated in real-life contexts. Learning was scaffolded using different teaching approaches such as podcasts, and experiential simulated learning, to offer learners multiple ways of engaging with content. Scaffolding is recognised as learning material being broken into smaller chunks of learning and in this way aligns with case-based learning [25]. In this way, we hoped that not only would students engage in problem-solving, and develop clinical decision-making skills [26, 27], but that they would also achieve deep and lifelong learning and ultimately have an ‘aha’ moment when it all made sense.

### Reflections on using an unfolding case study approach

Findings were divided, with some students enjoying the unfolding case studies and others describing them as not sufficiently challenging. The scaffolded learning approach that we developed incorporated a range of teaching approaches that enabled them to engage with the content in a way that fitted in with their lifestyle, even if the teaching method did not align with their individual learning preferences. Students reported differing views about the case studies; some enjoyed the unfolding nature while others wanted more context and direction to feel that they could make an informed clinical decision. Nonetheless, even though they did not like information being presented in smaller chunks one student recognised it meant they analysed the information they received more deeply.

Other learning tools such as podcasts were not always valued by participants and yet, the fact that students were able to provide feedback on their use does indicate that they at least attempted to engage with them.

Student reflections indicate that perhaps the use of unfolding case studies as a learning approach is not the solution to engagement, and that often more traditional teaching methods were preferred Indeed, Hobbs and Robinson’s [28] study of undergraduate nursing students in the US supported Carter and Welch’s [14] findings that the use of unfolding case studies were of no direct benefit, whilst Ellis et al.’s., [29] study confirmed that for final year nurse practitioner students unfolding case studies were beneficial in developing critical thinking and stimulating clinical reasoning. Considering these two conflicting findings, further consideration is needed of how to engage highly motivated GEN students.

As such, our results suggest it can be difficult to predict the needs of the GEN students given the diversity of their previous academic qualifications, career, and often significant life experience they bring to the programme [30, 31]. Interestingly students in this study simultaneously demonstrated insight into their needs supporting their previous academic study experience and felt sufficiently secure to voice them, which supports evidence found in D’Antonio et al.’s [32] study. This suggests that GEN students’ capabilities need to be embraced and incorporated when planning curriculum and scaffolding learning. Anecdotally, we have found that students embrace experiential learning such as that offered in simulation labs whether this involves the use of simulated manikins or not, it seems the hands-on learning offers not only the opportunity to experience simulated reality but also fosters collaboration and problem solving with peers that enables them to dwell in learning of what it is to be a nurse.

### Graduate entry students recognised as experienced learners

Our students were not overwhelmingly supportive of the pedagogical approach of unfolding case studies we adopted. As previously recognised GEN students are experienced learners and whilst having differing educational backgrounds bring individual experience and knowledge of their own approach to their learning. Nonetheless, the value of their previous learning experience appears problematic in that those learned behaviours and attitudes need to be refocused to engage with learning how to become a nurse, as demonstrated in the academic staff reflections. Despite this background experience and perceived confidence, some students reflected that online engagement that involved exploring the case studies in discussion forums with colleagues was uncomfortable. This was surprising to the academic staff and contrasted sharply with their reflections on the activity but has been previously noted by Boling et al., [33].

#### Implications

Given the disparity that exists between student and academic staff experiences, as demonstrated in our study, co-designing content delivery may offer a progressive solution. By engaging ‘students as partners’ it offers them a much deeper level of involvement in future teaching delivery through collaboration and reciprocation of ideas, thus culminating in appropriate curriculum design [34]. Collaborating with students in course design might facilitate students learning as they become cognisant of the active engagement of academic staff [9, 10, 35]. In the future, we aim to involve students in any curriculum review and course development to ensure their perspectives influence curriculum design and content delivery.

Even so, our initial intention of scaffolding learning by offering different ways for students to engage with content is supported by recent research by Dong et al. [36] who found that students performed better academically in a flipped classroom. This point, in association with our findings, suggests that the best approach to content delivery for graduate entry nursing students is to ensure students are involved in curriculum and course design alongside the delivery of learning experiences that are well facilitated and supported by faculty so that students are aware of the expectations, required of them, and importantly how they will be assessed.

### Limitations

We acknowledge that the sample size in this study is small in terms of generalisability. However, our findings offer interesting, detailed and in-depth insights into the experiences and needs of both GEN students and the academic staff involved in the development and delivery of educational material. Further work needs to be undertaken to evaluate the experiences of GEN students from a range of educational providers. A longitudinal study has been undertaken to explore the motivations and experiences of GEN students in Australasia [37], which will also support these findings regarding the learning needs of GEN students.

## Conclusion

This study has provided a platform through which academics and GEN students can share their insights of teaching and learning experiences. The results offer a clear insight into what these students expect and need to expedite their learning and how teaching staff must respond. While participants' views were somewhat mixed in relation to the use of unfolding case studies and scaffolded learning these results demonstrate how GEN students are aware of their personal ways of learning and how this translates in terms of education needs. The sharing of these experiences provides an insightful lens through which to re-evaluate pedagogical approaches for GEN students. As such, we suggest that to meet the needs of GEN student’s not only is a blended pedagogical approach appropriate but expanding education design boundaries further through a co-design focused approach to GEN programme design.

### Supplementary Information

Additional file 1.

## Data Availability

The datasets generated and analysed during the current study are not publicly available due privacy and ethical restrictions of the participants, but are available from the corresponding author on reasonable request.
