# Supplementary material for: Using unfolding case studies to develop critical thinking for Graduate Entry Nursing students: an educational design research study
Source: BMC Nurs. 2024 Jun 11;23:399. doi: 10.1186/s12912-024-02076-8 (PMC11165757; doi:10.1186/s12912-024-02076-8)
Supplement: Supplementary file 1 — Additional file 1. [file 12912_2024_2076_MOESM1_ESM.docx]

## Additional File 1

## Questions for student participants

1. Using an unfolding case study approach to clinical reasoning and decision-making, in what ways, if there were any, has this enhanced your learning to meet the learning outcomes of the course?
2. In what ways and to what end have you utilised the teaching and learning resources available for this course?
3. Does the use of podcasts for example, with information shared by professionals in their field, improve your learning?
4. What suggestions, if you have any, do you have that would improve the delivery of the case study approach?
